# Fiber2 and hexon genes are closely associated with the virulence of the emerging and highly pathogenic fowl adenovirus 4

**DOI:** 10.1038/s41426-018-0203-1

**Published:** 2018-12-05

**Authors:** Yuhan Zhang, Ruxin Liu, Kaiyue Tian, Zeng Wang, Xia Yang, Dongsheng Gao, Youming Zhang, Jun Fu, Hailong Wang, Jun Zhao

**Affiliations:** 1 College of Animal Science and Veterinary Medicine, Henan Agricultural University, Zhengzhou, 450002 China; 2 Shandong University–Helmholtz Institute of Biotechnology, State Key Laboratory of Microbial Technology, Shandong University, Qingdao, 266237 China

## Abstract

Since May 2015, outbreaks of hydropericardium-hepatitis syndrome (HHS) caused by fowl adenovirus 4 (FAdV-4) with a novel genotype have been reported in China, causing significant economic losses to the poultry industry. A previous comparative analysis revealed that highly virulent FAdV-4 isolates contain various genomic deletions and multiple distinct mutations in the major structural genes fiber2 and hexon. To identify the genes responsible for the virulence of HHS-associated novel FAdV-4 isolates, FAdV-4 infectious clones were constructed by directly cloning the whole genome of a highly pathogenic FAdV-4 isolate (CH/HNJZ/2015) and that of a nonpathogenic strain (ON1) into a p15A-cm vector using the ExoCET method. Subsequently, the fiber2, hexon, and 1966-bp fragment-replaced mutant/recombinant viruses were constructed using Redαβ recombineering and *ccdB* counter-selection techniques. The pathogenicity of the rescued viruses was compared with that of the rescued parent viruses rHNJZ and rON1 in 3-week-old SPF chickens. Chickens infected with the rescued viruses carrying the fiber2 and/or hexon gene of the HNJZ strain developed similar clinical signs to the natural infection, with distinctive gross lesions and characteristic histological signs indicative of HHS observed in sick/dead chickens. Our results clearly demonstrated that the virulence of the novel highly pathogenic FAdV-4 strain was independent of the 1966-bp deletion and that the fiber2 and hexon genes have crucial roles in FAdV-4 pathogenicity. The data presented in this report will provide further insights into the crucial factors determining the pathogenicity of FAdV strains. Furthermore, the infectious clones generated based on the FAdV-4 genome can be used as a platform for studies of gene function and for the development of recombinant vaccines.

## Introduction

Fowl adenovirus 4 (FAdV-4) is a member of the species Fowl aviadenovirus C, which belongs to the *Aviadenovirus* genus of the *Adenoviridae* family^1^. FAdV-4 is the predominant causative agent of hydropericardium-hepatitis syndrome (HHS), which is characterized by an accumulation of clear, straw-colored fluid in the pericardial sac and an enlarged and discolored liver with foci of haemorrhage and/or nephritis^1–7^. HHS primarily affects 3 to 6-week-old broiler chickens, causing between 20 and 80% mortality^8–10^. HHS was first reported in Pakistan in 1987, with outbreaks having been subsequently recorded in Asia and in South and Central America^8,11–14^. Since May 2015, outbreaks of HHS caused by FAdV-4 with a novel genotype have been reported in China^15–18^, causing significant economic losses to the poultry industry.

The virulence determinants of FAdV-4 and the molecular basis of HHS pathogenesis are currently unknown. The capsid of FAdV-4 primarily consists of three exposed structural proteins, the hexon, fiber, and penton base, with the fiber protein being noncovalently linked to the penton base^19^. There are two separate fiber-encoding genes in the FAdV-4 genome^20^. Molecular analyses are frequently performed using the major FAdV structural proteins with antigenic specificity encoded by the hexon and fiber genes^21,22^. However, the role of virulence determinants in both proteins has remained unclear, although it was speculated that the fiber protein and a 12-kb region containing open reading frames (ORFs) 19, 27, 29, and tandem repeat region E (TR-E) as potential virulence determinants for FAdV-8 and FAdV-4, respectively ^15,23,24^.

The results of our previous study showed that the highly virulent Chinese FAdV-4 isolates contain various genomic deletions, especially a 1966-bp deletion on the right end region of the genome. In addition, these isolates contain multiple distinct amino acid mutations in their major structural genes, fiber2 and hexon, compared with nonpathogenic FAdV-4 strains^18^. Because the role of most FAdVs as primary pathogens has not been clearly established, the factors determining FAdV pathogenicity are not yet clear. Different serotypes, and even strains of the same serotype, can vary in their ability to produce illness and death^25–27^. The objective of this study was to determine the role of hexon, fiber2 and the 1966-bp deletion in determining virulence of FAdV-4.

## Results

### Generation of FAdV-4 infectious clones (p15A-cm-HNJZ and p15A-cm-ON1) and fiber2, hexon- and 1966-bp fragment-replaced mutant viruses

**Fig. 1:**
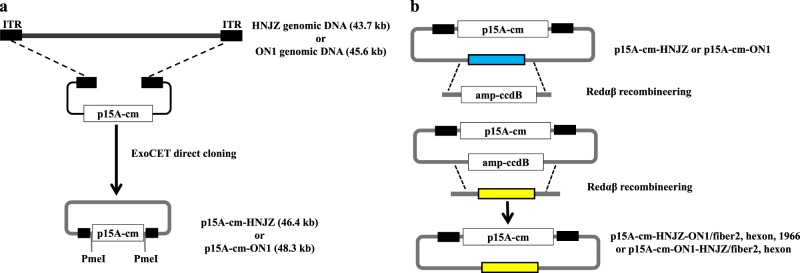
Construction of the fowl adenovirus 4 HNJZ/ON1 (FAdV-4 HNJZ/ON1) infectious clone by ExoCET direct cloning and generation of recombinant HNJZ/ON1 mutant viruses. **a** Direct cloning of the whole genomes of a highly pathogenic FAdV-4 isolate CH/HNJZ/2015 and a nonpathogenic strain ON1 into p15A-cm vectors using the ExoCET (Exonuclease Combined with RecET) method. ITR, inverted terminal repeat. **b** Generation of fiber2, hexon, and 1966-bp fragment-replaced mutant/recombinant viruses. Redαβ recombineering and *ccdB* counter-selection were used to seamlessly replace the fiber-2 and hexon genes and the 1966-bp deletion region of HNJZ in p15A-cm-HNJZ with their ON1counterparts and the fiber2 and hexon genes of ON1 in p15A-cm-ON1 with their HNJZ counterparts

The approach used to generate the FAdV-4 HNJZ and ON1 infectious clones and the fiber2, hexon, and 1966-bp fragment-replaced infectious clones is illustrated in Figs. 1a and b. The infectious clones of the highly virulent FAdV-4 strain HNJZ and the nonpathogenic strain ON1 were generated by directly cloning the viral genomic DNA into the p15A-cm vector using the ExoCET method^28^. Two *Pme*I restriction sites flanking the viral genomic DNA were integrated into oligonucleotides used to amplify the linear cloning vector. The correct infectious clones of p15A-cm-HNJZ and p15A-cm-ON1 were verified by restriction enzyme digestion (Supplementary Figure 1) and sequencing, after which they were linearized with *Pme*I and transfected into LMH cells. Cytopathic effects were observed at 5 to 6 days posttransfection, suggesting that the *Pme*I-digested infectious clones gave rise to viable viruses.

To generate the fiber2, hexon, and 1966-bp fragment-replaced recombinant viruses, Redαβ recombineering and *ccdB* counter-selection^29^ approaches were used to seamlessly replace the fiber2 and hexon genes and the 1966-bp deletion region of HNJZ in p15A-cm-HNJZ with their ON1counterparts, and the fiber2 or hexon gene of ON1 in p15A-cm-ON1 was replaced with its HNJZ counterpart. The *Bam*HI digestion banding patterns and sequencing were used to confirm the replacement/insertion and the identity of the infectious clones (Supplementary Figure 2). The resulting recombinant infectious clones p15A-cm-HNJZ-ON1/fiber2, p15A-cm-HNJZ-ON1/hexon, p15A-cm-HNJZ-ON1/1966, p15A-cm-ON1-HNJZ/fiber2, and p15A-cm-ON1-HNJZ/hexon were digested with *Pme*I and transfected into LMH cells to generate viable viruses named rHNJZ-ON1/fiber2, rHNJZ-ON1/hexon, rHNJZ-ON1/1966, rON1-HNJZ/fiber2, and rON1-HNJZ/hexon, respectively. The in vitro stability of rescued viruses after four passages was confirmed by PCR amplification and sequencing with specific primers flanking the replacement/insertion region (Data not shown).

### Viral growth kinetics

**Fig. 2:**
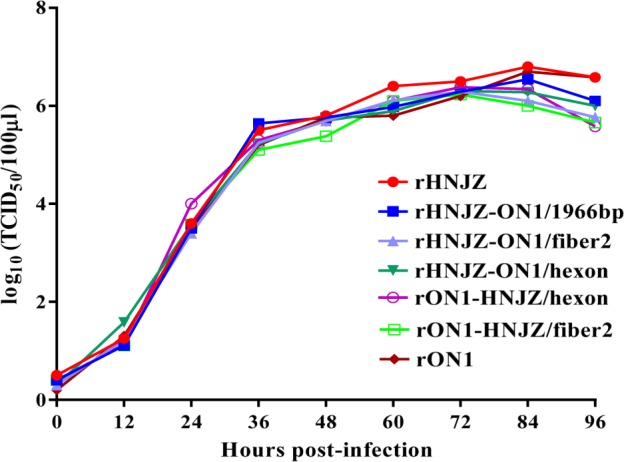
One-step growth curves. Leghorn male hepatocellular (LMH) cells were infected with rescued parent and mutant viruses at a multiplicity of infection of 0.001, and each virus was harvested at the indicated time points and titrated as described. Total viral titers were determined in three technical replicates and are expressed as the median tissue culture infective dose (TCID_50_/100 μl)

The growth kinetics of the rescued parent and mutant viruses were determined and compared. As shown in Fig. 2, relative to the rescued parent viruses rHNJZ and rON1, the rHNJZ-ON1/fiber2, rHNJZ-ON1/hexon, rHNJZ-ON1/1966, rON1-HNJZ/fiber2, and rON1-HNJZ/hexon viruses replicated in a similar manner, and no significant differences were noted among the growth curves, suggesting that these substitutions did not affect the replication of the rescued mutant viruses. The peak titer of each virus was 6.8 log_10_TCID_50_/100μl for rHNJZ, 6.54 log_10_TCID_50_/100μl for rHNJZ-ON1/1966, 6.29 log_10_TCID_50_/100 μl for rHNJZ-ON1/fiber2, 6.3 log_10_TCID_50_/100μl for rHNJZ-ON1/hexon, 6.7 log_10_TCID_50_/100 μl for rON1, 6.23 log_10_TCID_50_/100μl for rON1-HNJZ/fiber2, and 6.38 log_10_TCID_50_/100 μl for rON1-HNJZ/hexon, suggesting that all of the rescued viruses replicated at similar level in vitro.

### Clinical signs, gross pathology, and histology

**Fig. 3:**
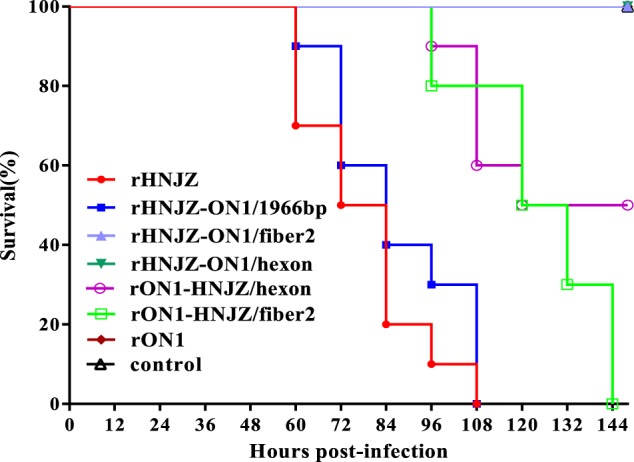
Survival rates of chickens after inoculation with different FAdV-4 viruses. Seven groups, each consisting of 10 3-week-old SPF chickens, were infected orally with 0.2 ml of 10^5^ TCID_50_ of each rescued FAdV-4 strain. One group of 10 chickens was left uninfected as control. The infected and control groups were separately housed in different negative-pressure isolators and monitored daily for 14 days, and the morbidity and mortality of animals were recorded

At 48 h.p.i, chickens infected with rHNJZ and rHNJZ-ON1/1966 developed similar clinical signs to a natural infection, characterized by depression and huddling together with ruffled feathers. The onset of mortality was recorded at 60 h.p.i. for chickens infected with rHNJZ and rHNJZ-ON1/1966, and there were no survivors at 108 h.p.i. (Fig. 3). At 80 h.p.i., chickens infected with rON1-HNJZ/fiber2 and rON1-HNJZ/hexon also developed similar clinical signs to a natural infection. The onset of mortality was recorded at 96 h.p.i. for chickens infected with rON1-HNJZ/fiber2 and rON1-HNJZ/hexon, and there were no survivors at 135 h.p.i. in rON1-HNJZ/fiber2-infected group. Chickens in the rON1-HNJZ/hexon-infected group exhibited a mortality of 50% throughout the experiment. No significant clinical symptoms or death were observed in the control group and groups infected with rHNJZ-ON1/fiber2, rHNIZ-ON1/hexon, or rON1.

**Fig. 4:**
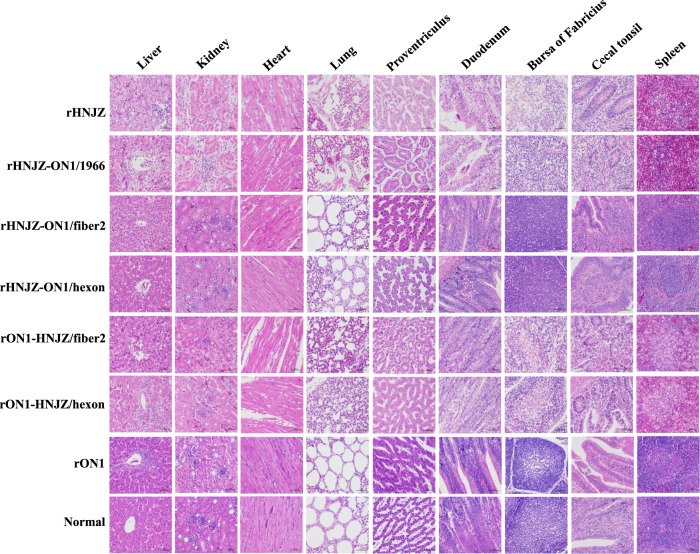
Representative histological changes in different tissues from chickens inoculated with rescued FAdV-4. Degeneration, necrosis of hepatocytes, and intranuclear inclusion bodies in hepatocytes; lymphocytic infiltrates in association with myocarditis; degeneration and vacuolar necrosis of renal tubular epithelium; glandular epithelial cell edema and necrosis in proventriculus; pulmonary interstitial edema and lymphocyte infiltration in the lungs; mucosal epithelial cell nuclear fragmentation, disintegration and necrosis, and intestinal villi necrosis in the duodenums; disintegration of lymphocytes of bursa of Fabricius; severe reduction and necrosis of lymphocytes in the spleens; partial necrosis of the lymphocytes in the lamina propria; and necrosis of the crypt epithelial cells in the cecal tonsils were detected in chickens infected with rHNJZ, rHNJZ-ON1/1966, rON1-HNJZ/fiber2, and rON1-HNJZ/hexon. No lesions were observed in the corresponding tissues of chickens in the control group or in the rON1, rHNJZ-ON1/hexon, and rHNJZ-ON1/fiber2 inoculated groups. (H&E stain, original magnification 400×, scale bar = 50 μm)

Distinctive gross lesions indicative of HHS, hydropericardium, and a discolored and swollen liver were observed for all sick/dead chickens. The characteristic histological signs of HHS, including degeneration, necrosis of hepatocytes, and intranuclear inclusion bodies in hepatocytes were also detected in chickens infected with rHNJZ, rHNJZ-ON1/1966, rON1-HNJZ/fiber2, and rON1-HNJZ/hexon. Other similar histological lesions presented by chickens in these groups included degeneration and vacuolar necrosis of renal tubular epithelium; glandular epithelial cell edema and necrosis in proventriculus; lymphocytic infiltrates in association with myocarditis; pulmonary interstitial edema and lymphocyte infiltration in the lungs; disintegration of lymphocytes in the bursa of Fabricius; a severe reduction and necrosis of lymphocytes in the spleens; partial necrosis of the lymphocytes in the lamina propria; and necrosis of the crypt epithelial cells in cecal tonsils. However, chickens in the control group and groups infected with rHNJZ-ON1/fiber2, rHNJZ-ON1/hexon, and rON1 did not present any gross lesions indicative of HHS (Fig. 4).

### Stability of the rescued virus in vivo

Total DNA was extracted from different tissues and cloacal swabs from dead or alive chickens, and the stability of the rescued viruses in vivo was verified by PCR and sequencing with primers flanking the insertion region (Supplementary Table 3). The sequencing results demonstrated that the recombinant regions in the rescued viruses were stable (Sequence data are available upon request).

### Viral genome copy number in tissues

**Fig. 5:**
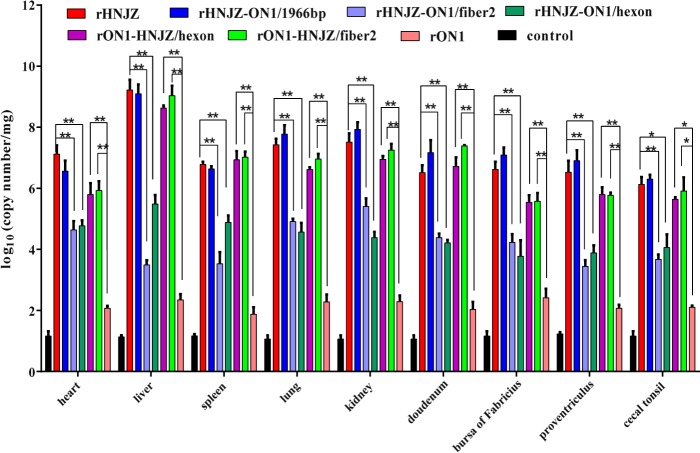
Viral loads in different tissues of chickens inoculated with rescued FAdV-4. Heart, liver, kidney, spleen, lung, proventriculus, duodenum, bursa of Fabricius, and cecal tonsil tissue samples of chickens in each group were collected from dead chickens during the experiment or euthanized chickens at the end of experiment. Viral loads in different tissues were determined by a SYBR Green I quantitative real-time PCR using FAdV-4 ORF14 gene as an indicator for the presence of viral DNA. The final concentration was calculated as copy numbers per milligram of tissue sample. Results are presented at the means ± standard error of mean. Asterisks (*) mark the viral loads that were significantly different between the groups (*p* < 0.05)

Viral genome copy numbers in heart, liver, kidney, spleen, lung, proventriculus, duodenum, bursa of Fabricius, and cecal tonsil tissue samples were determined by qRT-PCR, and the results are presented in Fig. 5. Only background levels of viral DNA were detected in tissues from chickens in the negative control group. The organ with the highest number of viral copies was the liver, followed by the kidney, lung and duodenum. Among the assayed tissues, those from the rHNJZ-infected chickens exhibited the highest number of viral copies, followed by the tissues from chickens infected with rHNJZ-ON1/1966, rON1-HNJZ/fiber2, rON1-HNJZ/hexon, rHNJZ-ON1/hexon, rHNJZ-ON1/fiber2, and rON1. Viral copy numbers in the tissues infected with the parent rHNJZ virus were significantly higher than that observed in the rHNJZ-ON1/hexon- and rHNJZ-ON1/fiber2-infected tissues (*p* < 0.01), and viral copy numbers in the rON1-HNJZ/fiber2 and rON1-HNJZ/hexon-infected tissues were significantly higher than that observed in the parent rON1-infected tissues (*p* < 0.01). The viral copy number in each tissue infected with rON1 was significantly lower than that observed in other viruses-infected tissues (*p* < 0.05). The tissues infected with viruses carrying the fiber2 and/or hexon gene of the highly virulent strain HNJZ (rHNJZ, rHNJZ-ON1/1966, rON1-HNJZ/fiber2, and rON1-HNJZ/hexon) exhibited much higher viral copy numbers than those of tissues infected with viruses carrying the fiber2 and/or hexon gene of a nonpathogenic strain ON1(rHNJZ-ON1/hexon, rHNJZ-ON1/fiber2, and rON1) (*p* < 0.05). It is worth noting that tissues and cloacal swabs from chickens infected with mutant viruses carrying only the fiber2 gene of the highly virulent strain HNJZ (rHNJZ-ON1/hexon and rON1-HNJZ/fiber2) exhibited higher viral copy numbers than those of samples from chickens infected with mutant viruses carrying only the hexon gene of the highly virulent strain HNJZ (rHNJZ-ON1/fiber2 and rON1-HNJZ/hexon), even though the difference was not significant.

### Viral shedding

**Fig. 6:**
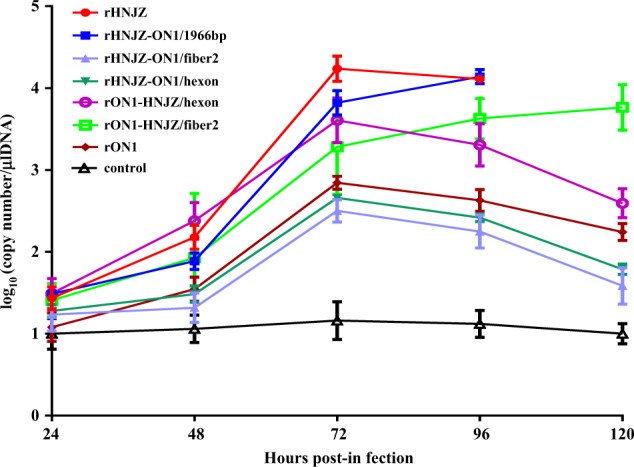
Viral shedding in chickens of different groups inoculated with rescued FAdV-4. Cloacal swabs were collected at 24, 48, 72, 96, and 120 h postinfection (h.p.i.) and their virus loads were determined by a SYBR Green I quantitative real-time PCR assay using the FAdV-4 ORF14 gene as an indicator for the presence of viral DNA. The final concentration was calculated as copy numbers per microliter of extracted DNA from cloacal swabs. The results are presented at the means ± standard error of mean

Chickens in all virus-infected groups excreted viruses until the termination of the experiment. As shown in Fig. 6, the peak titer occurred at 3 days postinfection (d.p.i.) for most groups, except for the rHNJZ-ON1/1966 and rON1-HNJZ/fiber2-inoculated groups, in which the titers of shed viruses increased throughout the experiment. Differences in the shed viral titers between the rHNJZ or rHNJZ-ON1/1966 groups and those of the other groups; between rHNJZ or rHNJZ-ON1/1966 and the rHNJZ-ON1/hexon or rHNJZ-ON1/fiber2 groups; between rON1 and the rON1-HNJZ/hexon or rON1-HNJZ/fiber2 groups; and between rON1-HNJZ/hexon or rON1-HNJZ/fiber2 and the rHNJZ-ON1/hexon or rHNJZ-ON1/fiber2 inoculated chickens were significant (*p* < 0.001) at 3 d.p.i. when evaluated by *t* tests. However, the difference in titers between the rON1-HNJZ/hexon- and rON1-HNJZ/fiber2-inoculated chickens and the rHNJZ-ON1/hexon and rHNJZ-ON1/fiber2-inoculated chickens was not significant (*p* > 0.05) at 3 d.p.i. No viral excretion was detected in any of the samples taken from the negative control animals. Chickens inoculated with the parent strain HNJZ exhibited the highest peak in shed viral titer, followed by rHNJZ-ON1/1966, rON1-HNJZ/hexon, rON1-HNJZ/fiber2, rON1, rHNJZ-ON1/hexon, and rHNJZ-ON1/fiber2.

## Discussion

FAdV-4 is the predominant etiological agent of HHS, which is an economically important disease in the poultry industry. Owing to the limited available information on the complete genome sequences of FAdV-4 strains with different pathogenicity, the difficulty in manipulating the large size of the FAdV-4 genome and a shortage of reverse genetic systems for FAdV-4, the factors determining FAdV-4 pathogenicity profiles remain unclear. Recent studies of fully sequenced genomes of pathogenic and nonpathogenic FAdV-4 isolates identified potential genomic variations associated with virulence. The primary variations include nucleic acid insertions and deletions in ORFs 19, 27, 48, and 19 A and amino acid substitutions and deletions in the major structural proteins fiber2 and hexon^18^. In this study, to elucidate the role of these variations in FAdV-4 pathogenicity, FAdV-4 infectious clones were constructed by directly cloning the whole genome of a highly pathogenic FAdV-4 isolate (CH/HNJZ/2015) or that of a nonpathogenic strain (ON1) into a p15A-cm vector using the ExoCET method^28^. The established FAdV-4 reverse genetic systems allowed us to systematically study the potential functions of these variations and their association with virulence. By taking advantages of the Redαβ recombineering and *ccdB* counter-selection techniques, the fiber2, hexon, and 1966-bp fragment-replaced FAdV-4 mutants/recombinants were constructed by seamlessly replacing the fiber2 and hexon genes and the 1966-bp deletion region of HNJZ in p15A-cm-HNJZ with their ON1 counterparts and the fiber2 and hexon genes of ON1 in p15A-cm-ON1 with their HNJZ counterparts. This process produced a pure population of recombinant virus for each construct. PCR amplification and sequencing with specific primers flanking the replacement/insertion region confirmed the stability of the recombinants. These results demonstrated the efficacy and utility of p15A-cm-HNJZ and p15A-cm-ON1 as infectious clones for molecular studies and the rapid generation of FAdV-4-based recombinant vaccines.

All of the rescued viruses exhibited similar replication pattern to the corresponding parent viruses in vitro, suggesting that the DNA manipulations in this study did not affect the replication of the rescued mutant viruses. The sequencing results demonstrated that the recombinant regions in the rescued viruses were genetically stable after four serial passages, demonstrating that the rescued viruses can be used for pathogenicity assessments. The virulence of the recombinant viruses was tested in SPF chickens and compared with that of the rescued parental viruses. Chickens inoculated with the parental rHNJZ exhibited 100% mortality, reconfirming the high virulence of the virus that we previously observed^18^. Chickens inoculated with rHNJZ-ON1/1966 also exhibited 100% mortality, indicating that the natural 1966 bp-deletion region in the genome of the highly virulent FAdV-4 novel genotype is dispensable for viral replication and virulence and is suitable as an insertion site for foreign genes.

Virulence testing of the recombinant viruses rHNJZ-ON1/hexon, rHNJZ-ON1/fiber2, rON1-HNJZ/hexon, and rON1-HNJZ/fiber2 indicated that hexon and fiber2 are responsible for differences in virulence between the highly virulent strain HNJZ and the nonpathogenic strain ON1. Chickens inoculated with rON1-HNJZ/fiber2 also exhibited 100% mortality at 144 h.p.i., while those inoculated with rON1-HNJZ/hexon only exhibited 50% mortality by the termination of the experiment. These differences in mortality patterns suggest that fiber2 plays a greater role than hexon in FAdV-4 pathogenicity. Unexpectedly, no clinical symptoms indicative or death were observed in the groups infected with rHNJZ-ON1/fiber2 or rHNJZ-ON1/hexon, even though rHNJZ-ON1/fiber2 and rHNJZ-ON1/hexon still carried the hexon or fiber2 gene from the HNJZ strain. These changes were consistent with the observations that viral loads in tissues and the viral shedding levels in groups infected with rHNJZ-ON1/fiber2, rHNIZ-ON1/hexon, or the parent rON1 were much lower than those of other virus-infected groups. This observation suggests that viruses carrying the hexon or fiber2 gene from nonpathogenetic ON1 replicate more slowly in vivo, even though the in vitro growth capacity of these viruses is similar. Differences in knob domain of the fiber gene as well as in L1 loop domain of the hexon gene have been implicated in the differences in tissue tropism and virulence for human and canine adenoviruses^30–33^. As previously stated, when comparing the amino acid sequences of hexon and fiber2 with those in the nonpathogenic strain ON1, highly virulent strains, including the HNJZ strain evaluated in this study, exhibit amino acid changes of 164 S, 188 R, 193 R, 195Q, 238D, 240 T, 243 N, 263I, and 264 V in the L1 loop region of the hexon protein and 219D, 232Q, 300 T, 305 A, 307 A, 329 L, 378 T, 380 T, 435 S and 453 A in the fiber2 protein. The fiber2 protein of CH/HNJZ/2015 contains 479 amino acids residues, 5 amino acids (ENGKP at 11–15) more than ON1 in the tail region. Previous model indicated that the fiber protein consists of an N-terminal tail region, involved in attachment to the penton base, a shaft region that appears to consist of multiple copies of a repeating motif of approximately 15 amino acids, and a knob that is involved in attachment of the virus to the host cell^24^. Our results suggest that changes in the tail and knob regions result in different binding affinities toward host cell receptors and affect viral infectivity in chickens^34^.

The gross and histological lesions were observed in chickens infected with viruses carrying the fiber2 and/or hexon gene from the highly virulent strain HNJZ. Severe lesions were observed in chickens infected with the rHNJZ, rHNJZ-ON1/1966, and rON1-HNJZ/fiber2. In contrast, lesions were not visible in chickens infected with rON1, rHNJZ-ON1/hexon, and rHNJZ-ON1/fiber2 and in the negative control groups. Tissues and cloacal swabs from chickens infected with mutant viruses carrying only the fiber2 gene of the highly virulent strain HNJZ (rHNJZ-ON1/hexon and rON1-HNJZ/fiber2) exhibited higher viral copy numbers than those of samples from chickens infected with mutant viruses carrying only the hexon gene of the highly virulent strain HNJZ (rHNJZ-ON1/fiber2 and rON1-HNJZ/hexon), even though the difference was not significant. These results reconfirmed that fiber2 plays a greater role than hexon in FAdV-4 pathogenicity. The fiber protein is of great importance for virus-cell interactions, as demonstrated for FAdV-1^35^, and these results may also explain the importance of the fiber protein as a virulence factor^24,25,36,37^. It is interesting to note that hexon has also been implicated in pathogenicity, as a previous study showed that changes in the hexon gene are involved in attenuation of fowl adenovirus^38^.

FAdVs are a poorly characterized group for which very little is known regarding the determinants of virulence. As a number of FAdV-4 isolates exhibiting differences in virulence are available, FAdV-4 is a useful model for studying FAdV virulence, for which the infectious clones of highly virulent and nonpathogenetic FAdV-4 strains established in this study will be powerful tools. To the best of our knowledge, the genes responsible for differences in the virulence of FAdV-4 strains were identified for the first time in this study. Furthermore, our results also raise interesting questions concerning some other facets of FAdV-4 biology. In future studies,, the domains in fiber2 and hexon that are crucial in determining FAdV-4 pathogenicity will be investigated further.

In summary, our results demonstrated that the virulence of the novel highly pathogenic FAdV-4 is independent of the 1966-bp deletion, and the fiber2 and hexon genes play crucial roles in FAdV-4 pathogenicity. The data presented in this report provide insights into the crucial factors determining the pathogenicity of FAdV strains, and the infectious clones developed based on the FAdV-4 genome can be used as a platform for gene function studies and for the development of recombinant vaccines.

## Materials and methods

### Viruses and cells

The highly virulent FAdV-4 strain CH/HNJZ/2015 (GenBank accession No. KU558760) was isolated from the livers of infected birds exhibiting clinical signs of HHS in Henan province, China, as previously described^18^. The nonpathogenic FAdV-4 strain ON1^20^ (GenBank accession No.GU188428) was kindly provided by Dr. Éva Nagy from the Department of Pathobiology, University of Guelph, Guelph, Canada. The FAdV-4 HNJZ, ON1 and rescued viral strains were plaque-purified, propagated and titrated continuously in leghorn male hepatocellular (LMH) cells (ATCC, CRL-2117) as previously described^39^.

### *E. coli* strains and plasmids

*E. coli* GB05-dir harboring pSC101-BAD-ETgA-tet was used for direct cloning of genomic DNA from the FAdV-4 HNJZ or ON1 strain^28^. *E. coli* GBred-gyrA462 and GB05-red were used for seamless substitution of the fiber2 and hexon genes and the 1966-bp deletion region of CH/HNJZ/2015 and ON1^29^. p15A-cm-tetR-tetO-*ccdB*-hyg was used as template to amplify the p15A-cm vector for direct cloning^40^, and p15A-amp-*ccdB* was used as template to amplify the amp-*ccdB* cassette^29^.

### Construction of FAdV-4 HNJZ and ON1 infectious clones

FAdV-4 genomic DNA was extracted from lysates LMH cells infected with the highly pathogenic isolate CH/HNJZ/2015 or the nonpathogenic strain ON1 using a QIAamp DNA Blood Mini Kit (Qiagen, Hilden, Germany) according to the manufacturer’s instructions. The p15A-cm linear cloning vector was purified with gel electrophoresis after PCR amplification using the oligonucleotides listed in Supplementary Table 1. The genomic DNA and p15A-cm linear vector were treated with T4 DNA polymerase according to the described protocol^28^, and electroporated into L-arabinose induced *E. coli* GB-05 dir harboring pSC101-BAD-ETgA-tet. The correct infectious clones of p15A-cm-HNJZ and p15A-cm-ON1 generated by homologous recombination were identified with *EcoR*I and *Ase*I digestion analysis of colonies on LB plates supplemented with chloramphenicol.

### Construction of the fiber2-, hexon- and 1966-bp fragment-replaced mutant/recombinant viruses

To identify the genes associated with virulence, fiber2, hexon, and 1966-bp fragment-replaced mutant/recombinant viruses were constructed. Redαβ recombineering and *ccdB* counter-selection were used to seamlessly replace the fiber2 and hexon genes and the 1966-bp deletion region of HNJZ on p15A-cm-HNJZ with their ON1 counterparts and the fiber2 and hexon genes of ON1 on p15A-cm-ON1 with their HNJZ counterparts according to a previously described protocol^29^. Briefly, the *amp*-*ccdB* cassettes and relevant genes were PCR amplified using the oligonucleotides listed in Supplementary Table 2. The target genes in p15A-cm-HNJZ or p15A-cm-ON1 were first replaced with the *amp*-*ccdB* cassette using linear-circular homologous recombination (LCHR) mediated by Redαβ recombinases expressed in *E. coli* GBred-gyrA462, which carries an Arg462Cys mutation in the GyrA subunit of DNA gyrase that confers CcdB resistance. Next, the *amp*-*ccdB* cassette was replaced with the gene of interest using LCHR mediated by the Redαβ recombinases expressed in *E. coli* GB05-red, which is sensitive to the CcdB toxin. The correct infectious clones were verified by restriction enzyme digestion and sequencing. The *Pme*I-digested infectious clones were transfected into LMH cells using Lipofectamine 2000 (ThermoFisher Scientific, Shanghai, China) to generate the recombinant viruses rHNJZ-ON1/fiber2, rHNJZ-ON1/hexon, rHNJZ-ON1/1966, rON1-HNJZ/fiber2, rON1-HNJZ/hexon, rHNJZ and rON1. The stability of the recombinant regions over four passages was verified by PCR and sequencing with primers flanking the insertion sites (Supplementary Table 3).

### One-step growth curves

The in vitro replication capacity of the rescued viruses was assessed in LMH cells. Briefly, LMH cell monolayers (1.3 × 10^6^ cells/well) in 6-well plate were infected with the fourth passage of rHNJZ, rHNJZ-ON1/fiber2, rHNJZ-ON1/hexon, rHNJZ-ON1/1966, rON1, rON1-HNJZ/fiber2, or rON1-HNJZ/hexon at 2 × 10^3^ TCID_50_ (multiplicity of infection≈0.001), and each virus was harvested at the indicated times postinfection (p.i.). The median tissue culture infective dose (TCID_50_) of the harvested viruses was determined using 96-well plates according to previously reported methods with minor modification^41^. Briefly, LMH cells in 96-well plates were inoculated with 10-fold dilutions of viral stocks (from 10^–1^ to 10^–11^) in triplicate and incubated at 37 ℃ with 5% CO_2_ for 6 days. The cytopathic effect was observed using a microscope, and the TCID_50_ values were calculated according to the Reed and Muench method^42^.

### Pathogenicity assessment

The pathogenicity of the rescued mutant viruses was compared with the rescued parent viruses rHNJZ and rON1 in 3-week-old SPF chicks (Poultry Institute, Shandong Academy of Agricultural Science). The protocol was approved by the Animal Care and Use Committee of Henan Agricultural University (Henan, China) and was performed in accordance with the “Guidelines for Experimental Animals’ of the Ministry of Science and Technology (Beijing, China). Seven groups, each consisting of ten 3-week-old SPF chickens, were infected orally with 0.2 ml of 10^5^ TCID_50_ of the fourth passage of each rescued virus. One group of 10 chickens remained uninfected as a control. The infected and control groups were separately housed in different negative-pressure isolators and monitored daily for 14 days. Heart, liver, kidney, spleen, lung, proventriculus, duodenum, bursa of Fabricius, and cecal tonsil tissue samples of chickens in each group were collected from dead chickens during the experiment or euthanized chickens at the end of experiment. Each organ was sectioned into two portions, one of which was fixed with 10% formalin, cut into sections and stained with hematoxylin-eosin, while the other section was stored at −80 °C for quantification of viral loads by SYBR Green I quantitative real-time polymerase chain reaction (qRT-PCR). Cloacal swabs were collected at 24, 48, 72, 96, and 120 h postinfection (h.p.i.) and their viral loads were also determined by SYBR Green I qRT-PCR. Pathogenicity was evaluated by observation of clinical signs as well as gross and histological lesions. The morbidity and mortality of animals was recorded.

### Stability of the rescued viruses in vivo and quantification of viral DNA in tissues and cloacal swabs

Total DNA was extracted from different tissues and cloacal swabs with a DNeasy Blood & Tissue Kit (Qiagen, Hilden, Germany). The stability of the rescued viruses in vivo was verified by PCR and sequencing with primers flanking the insertion region (Supplementary Table 3), and the viral loads were determined by SYBR Green I qRT-PCR performed with a LightCycler 1.5 (Roche, Basel, Switzerland). The FAdV-4 ORF14 gene was used as an indicator for the presence of viral DNA. The forward and reverse primers used were 5′-AGTGTGTATGTGCGTTGGGTAG-3′ and 5′-CATTGTCATAACGATGGTGTAG-3′, respectively^39^. The primers were universal for FAdV-4, generating a 136-bp amplification product. Each qRT-PCR reaction mixture contained 10 μl of 2 × SYBR Green Premix ExTaq II (TaKaRa Biotech, Dalian, China), 0.4 μl of each primer (25 μM) and 2 μl of the plasmid template in a total volume of 20 μl. The reaction conditions included a predenaturation step at 95 ℃ for 10 min followed by amplification for 40 cycles of a melting step at 95 ℃ for 10 s and an elongation at 60 ℃ for 1 min. The amplified 136-bp fragment was purified and cloned into the vector pMD18-T (TaKaRa Biotech, Dalian, China) to prepare a standard recombinant plasmid. Serial dilutions of the plasmid standard (from 10^–2^ to 10^–8^ copies/μl) were used to optimize the qRT-PCR reaction and to establish the standard curve. The standard and melting curves were established using Light Cycler version 3.5 (Roche). The final concentrations were calculated as copy numbers per milligram of tissue samples or per microliter of DNA. The results are presented at means ± standard error of mean.

### Statistical analysis

Statistical analyses were performed using the Multiple *t* test within the GraphPad Prism software package. For analyses of the viral loads in tissues, tests were performed between groups of chickens infected with each mutant and the corresponding parent virus. Significant differences were determined as *p < 0.05 (significant) or **p < 0.01 (highly significant).

## Electronic supplementary material

Table S1

Table S2

Table S3

Figure S1

Figure S2
