# Supplementary material for: Fiber2 and hexon genes are closely associated with the virulence of the emerging and highly pathogenic fowl adenovirus 4
Source: Emerg Microbes Infect. 2018 Dec 5;7:199. doi: 10.1038/s41426-018-0203-1 (PMC6279807; doi:10.1038/s41426-018-0203-1)
Supplement: Supplementary file 1 — Table S1 [file 41426_2018_203_MOESM1_ESM.docx]

**Table S1.** Oligonucleotides used for direct cloning of the FAdV4 genomic DNA.

| **Name** | **Sequence (5’-3’)** | **Templates** |
| --- | --- | --- |
| FAdV4-1 | CGCGCTGCGCGCGGCGGTTGTAAGTGTGTCAAAAGACGCGGTTATATAAGATGATGGTTTAAACAGATCCGAAAACCCCAAGTTACG | p15A-cm-tetR-tetO-hyg-ccdB |
| FAdV4-2 | CGCGCTGCGCGCGGCGGTTGTAAGTGTGTCAAAAGACGCGGTTATATAAGATGATGGTTTAAACAGATCCTTTCTCCTCTTTAGATC |  |
