# Supplementary material for: Fiber2 and hexon genes are closely associated with the virulence of the emerging and highly pathogenic fowl adenovirus 4
Source: Emerg Microbes Infect. 2018 Dec 5;7:199. doi: 10.1038/s41426-018-0203-1 (PMC6279807; doi:10.1038/s41426-018-0203-1)
Supplement: Supplementary file 2 — Table S2 [file 41426_2018_203_MOESM2_ESM.docx]

**Table S2.** Oligonucleotides used for seamless mutagenesis.

| **Name** | **Sequence (5’-3’)** | **Templates** |
| --- | --- | --- |
| ampccdBfiber2HNJZ -1 | TCCTATCCCTTTTTCCTATCAGGGTTACGTCTACTCCCCCAACGGGAACATTAATTAATTTGTTTATTTTTCTAAATAC | p15A-ampccdB |
| ampccdBfiber2HNJZ -2 | GCAATCAACGTTCATGACTCTTTATTTGACACGCGGTGGGGAGGGCGCGCTTAATTAATTTGTTCAAAAAAAAGCCCGC |  |
| fiber2ON1-1 | TCCTATCCCTTTTTCCTATCAGGGTTACGTCTACTCCCCCAACGGGAACAATGCTCCGAGCCCCTAAAAG | ON1 genomic DNA |
| fiber2ON1-2 | GCAATCAACGTTCATGACTCTTTATTTGACACGCGGTGGGGAGGGCGCGC |  |
| ampccdBhexonHNJZ -1 | ACTAAACGGGTTGTGTATGTATGTCGCGTTTCGTCTAGGTTCGCACCGCCTTAATTAATTTGTTTATTTTTCTAAATAC | p15A-ampccdB |
| ampccdBhexonHNJZ -2 | GTCCCGCAACTGAGACTCCGTGGTCCCCGTCATGCCGACGCTCTAAGGGTTTAATTAATTTGTTCAAAAAAAAGCCCGC |  |
| HexonON1-1 | ACTAAACGGGTTGTGTATGTATGTCGCGTTTCGTCTAGGTTCGCACCGCCATGGCGGCCCTCACGCCCG | ON1 genomic DNA |
| HexonON1-2 | GTCCCGCAACTGAGACTCCGTG |  |
| ampccdB1966HNJZ -1 | AACATAAGAATCAGGGGTGGCCCGTATACTAATCCCGTCACTGACGACACTTAATTAATTTGTTTATTTTTCTAAATAC | p15A-ampccdB |
| ampccdB1966HNJZ -2 | CACTCGAGAAGGAGCCTCTGAGCCGTACTCTATGCATTGCGTGATTGTGGTTAATTAATTTGTTCAAAAAAAAGCCCGCTC |  |
| 1966ON1-1 | AACATAAGAATCAGGGGTGGCCCGTATACTAATCCCGTCACTGACG | ON1 genomic DNA |
| 1966ON1-2 | CACTCGAGAAGGAGCCTCTGAG |  |
| ampccdB16HNJZ -1 | CCATGTCGGCCCTAATCGCCTCCGCAGCCGATACCGTCTCCGCCAGCGGATTAATTAATTTGTTTATTTTTCTAAATAC | p15A-ampccdB |
| ampccdB16HNJZ -2 | ATTGTTCCCGTTGGGGGAGTAGACGTAACCCTGATAGGAAAAAGGGATAGTTAATTAATTTGTTCAAAAAAAAGCCCGCTC |  |
| ampccdBfiber2ON1-1 | TATCCCTTTTTCCTATCAGGGTTACGTCTACTCCCCCAACGGGAACAACCTTAATTAATTTGTTTATTTTTCTAAATAC | p15A-ampccdB |
| ampccdBfiber2ON1-2 | GCAATCAACGTTCATGACTCTTTATTTGTCACGCGGTGGGGAGGGCGCGCTTAATTAATTTGTTCAAAAAAAAGCCCGCTC |  |
| fiber2HNJZ -1 | TATCCCTTTTTCCTATCAGGGTTACGTCTACTCCCCCAACGGGAACAACCATGCTCCGGGCCCCTAAAAGAAGAC | HNJZ genomic DNA |
| fiber2HNJZ -2 | GCAATCAACGTTCATGACTCTTTATTTGTCACGCGGTGGGGAGGGCGCGCTTACGGGAGGGAGGCCGCTGG |  |
| ampccdBhexonON1-1 | ACTAAACGGGTTGTGTATGTATGTCGCGTTTCGTCTAGGTTCGCACCGTCTTAATTAATTTGTTTATTTTTCTAAATAC | p15A-ampccdB |
| ampccdBhexonON1-2 | GTCCCGCAACTGAGACTCCGTGGTCCCCGTCATGCCGACGCTCTAAGGGTTTAATTAATTTGTTCAAAAAAAAGCCCGCTC |  |
| hexonHNJZ -1 | ACTAAACGGGTTGTGTATGTATGTCGCGTTTCGTCTAGGTTCGCACCGTCATGGCGGCCCTCACGCCCGAC | HNJZ genomic DNA |
| hexonHNJZ -2 | GTCCCGCAACTGAGACTCCGT |  |
