# Supplementary material for: Fiber2 and hexon genes are closely associated with the virulence of the emerging and highly pathogenic fowl adenovirus 4
Source: Emerg Microbes Infect. 2018 Dec 5;7:199. doi: 10.1038/s41426-018-0203-1 (PMC6279807; doi:10.1038/s41426-018-0203-1)
Supplement: Supplementary file 3 — Table S3 [file 41426_2018_203_MOESM3_ESM.docx]

**Table S3.** Oligonucleotides used for sequencing

| **Name** | **Sequence (5’-3’)** | **Templates** |
| --- | --- | --- |
| fiber2-seq-5’ | CACCGCCACCGGAAACGAGA | p15A-cm-HNJZ-ON1/fiber2  or p15A-cm-ON1-HNJZ/fiber2 |
| fiber2-seq-3’ | GGCCGAATTCCTGATGGTAG |  |
| hexon-seq-5’ | GGTGAACCCTCCCATCACGT | p15A-cm-HNJZ-ON1/hexon  or p15A-cm-ON1-HNJZ/hexon |
| hexon-seq-3’ | ACTCCAACAGTTTCTTGTCG |  |
| 1966-seq-5’ | ACCGAACTCACCGATTCAGC | p15A-cm-HNJZ-ON1/1966 |
| 1966-seq-3’ | GGTGTGGCGTTAATGATTAA |  |
