# Supplementary figures and images for: Fiber2 and hexon genes are closely associated with the virulence of the emerging and highly pathogenic fowl adenovirus 4
Source: Emerg Microbes Infect. 2018 Dec 5;7:199. doi: 10.1038/s41426-018-0203-1 (PMC6279807; doi:10.1038/s41426-018-0203-1)

## Slide 1
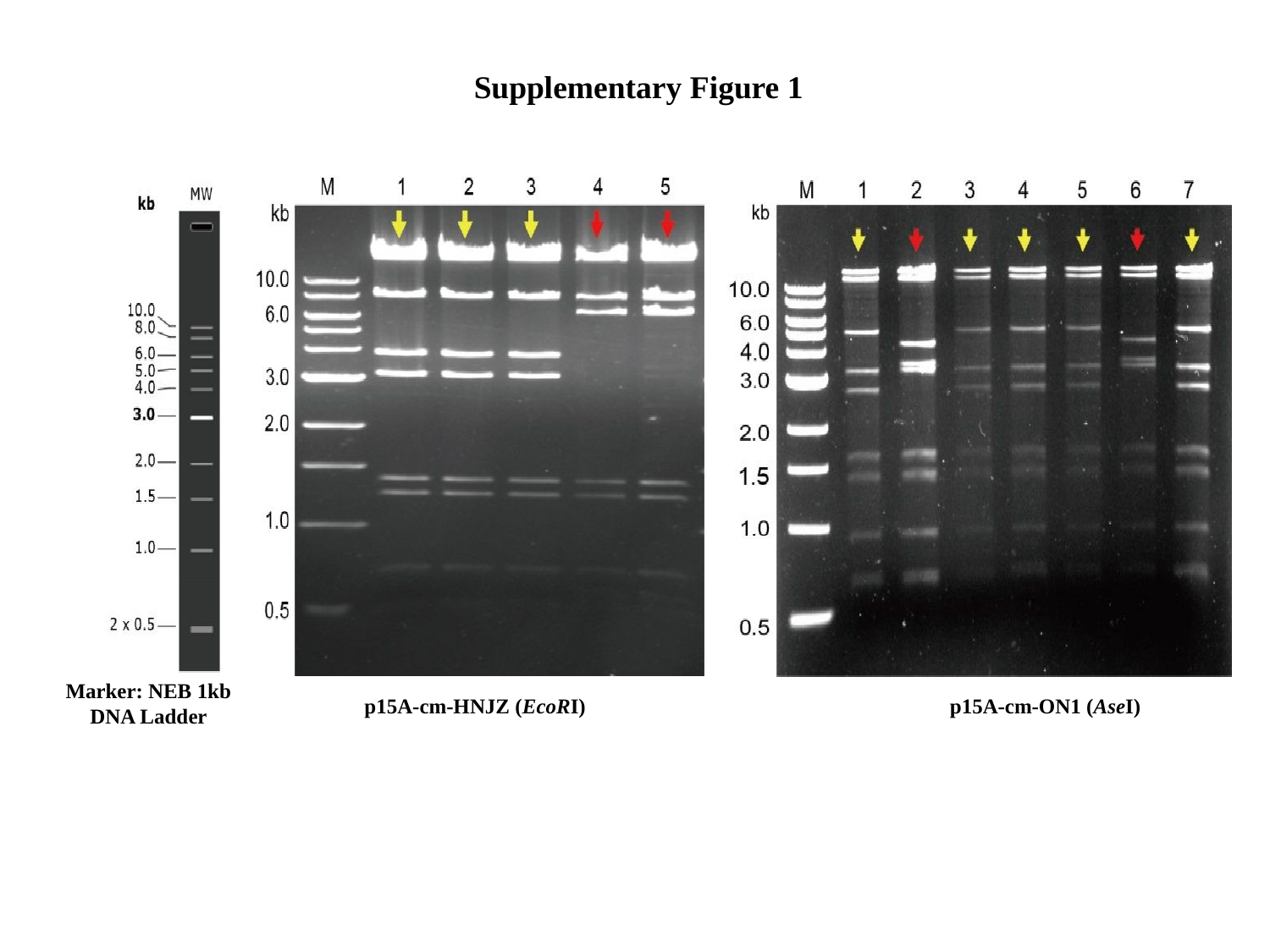

Supplementary Figure 1
Marker: NEB 1kb DNA Ladder
p15A-cm-HNJZ (EcoRI)
p15A-cm-ON1 (AseI)

Supplement: Supplementary file 4 — Figure S1 [file 41426_2018_203_MOESM4_ESM.pptx]

## Slide 1
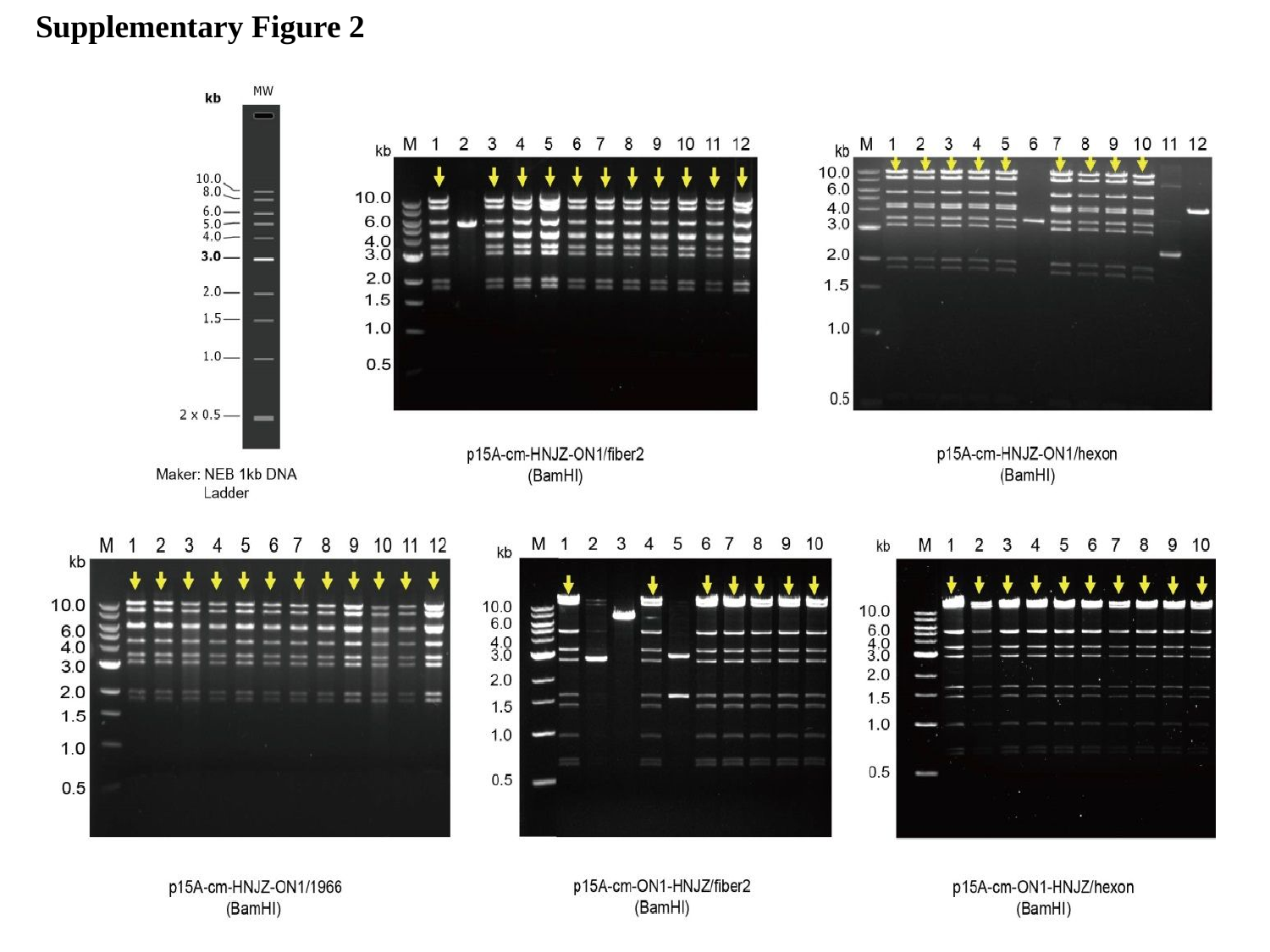

Supplementary Figure 2

Supplement: Supplementary file 5 — Figure S2 [file 41426_2018_203_MOESM5_ESM.pptx]
